# Measurement of a Preresonant Hyper-Raman Hyperpolarizability

**DOI:** 10.1021/acs.jpclett.6c00156

**Published:** 2026-02-26

**Authors:** Ryan P. McDonnell, Daniel D. Kohler, Wei Zhao, John C. Wright

**Affiliations:** † Department of Chemistry, 5228 University of Wisconsin−Madison, Madison, Wisconsin 53706, United States; ‡ School of Physical Sciences, 53678 University of Arkansas, Little Rock, Arkansas 72204, United States

## Abstract

Four wave mixing (FWM) spectroscopy is used to measure a preresonant hyper-Raman hyperpolarizability. The four wave mixing process known as hyper-difference frequency generation (HDFG), which depends upon the hyper-Raman hyperpolarizability, can be measured simultaneously with other well-studied four-wave mixing processes, which serve as an internal standard. We use coherent anti-Stokes Raman (CARS) response from deuterobenzene to quantify the acetonitrile ν­(CN) hyper-Raman hyperpolarizability. This internal standard method is applicable to any infrared active vibration and greatly diminishes the barriers to quantification of hyper-Raman hyperpolarizabilities.

## Graphical abstract

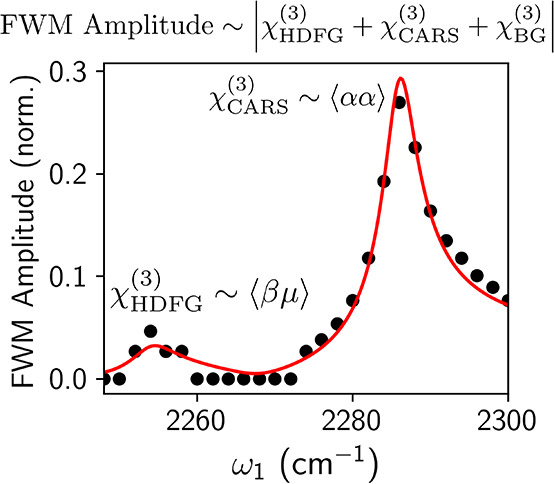

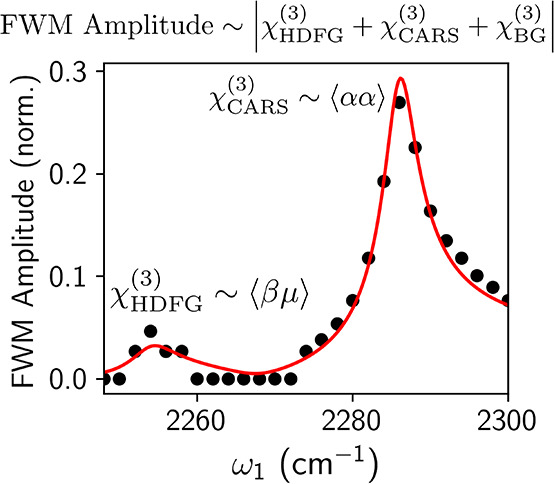

Developments in nonlinear spectroscopy and laser technology have made coherent nonlinear spectroscopies involving hyper-Raman transitions viable.^1–10^ Similarly, spontaneous hyper-Raman studies have had a resurgence in recent years.^11–19^ Hyper-Raman scattering, the two photon nonlinear analogue of Raman scattering,^20^ inelastically scatters light from frequency ω_*m*_ + ω_*n*_,^21^ usually with ω_*m*_ + ω_*n*_ = 2ω.^22^ Spontaneous hyper-Raman scattering is a fifth order process that is often weak, but usually performed with 2ω on resonance with an excited electronic state to amplify output.^23–25^ Hyper-Raman transitions can be useful for investigating non-Condon effects, solvent induced symmetry breaking, molecular orientation dynamics, among others.^6,17,21,23–33^ Hyper-Raman excitation spectra can investigate excited state surfaces and vibronic coupling schemes.^28^ Similar to how nonlinear spectroscopies that involve Raman transitions have a dependence on the Raman polarizability α,^34–36^ hyper-Raman based methods have a dependence on the hyper-Raman hyperpolarizability β.^32,37^ Nonlinear processes of particular interest that involve Raman or hyper-Raman transitions are coherent anti-Stokes Raman spectroscopy (CARS, Figure 1 left), which involves two Raman transitions,^38^ and hyper-difference frequency generation (HDFG, Figure 1 right), which involves an infrared and hyper-Raman transition.^39^

**Figure 1:**
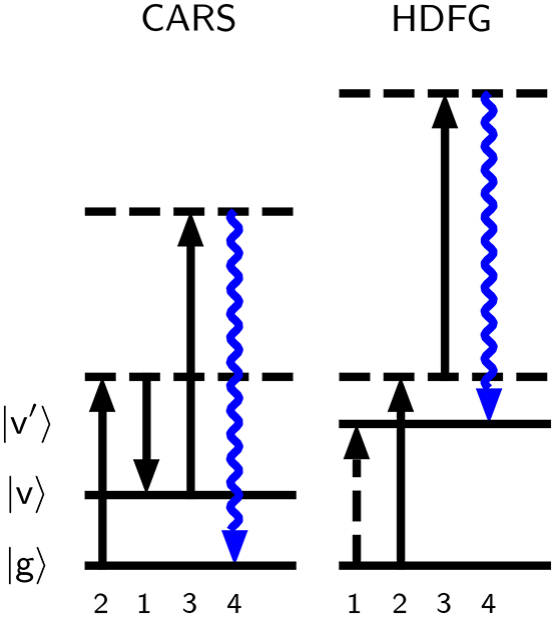
Wave Mixing Energy Level (WMEL) diagrams^70^ of coherent anti-Stokes Raman spectroscopy (CARS) and hyper-difference frequency generation (HDFG), the relevant singly resonant four wave mixing processes in the **k**_4_ = −**k**_1_ + **k**_2_ + **k**_3_ phase-matching geometry considered in this work.^2^ Solid (dashed) vertical arrows indicate a ket (bra) side transition. Solid (dashed) horizontal lines indicate a real (virtual) state.

The measurement of Raman cross-sections, while tedious, have been performed for numerous systems with different methods,^40–49^ which allows α to be measured with high accuracy. By measuring Raman cross-sections at multiple frequencies and fitting to well-known expressions from Albrecht theory,^50^ these measurements allow solvent Raman response to be used as internal standards and enable quantitative frequency-dependent resonance Raman spectroscopy, which has evolved to be a useful probe of molecular structure and excited state surfaces.^28,51–57^ These measurements have also permitted quantitative assessments of nonlinear susceptibilities, and thus the strength of nonlinear processes.^2,35,48,58–68^ However, while Raman cross-sections have been measured for numerous systems using multiple established methods, quantitative measurement of hyper-Raman cross-sections is notoriously difficult and has been limited to only a few systems.^25,27,69^ To date, two methods have been used to measure hyper-Raman scattering cross-sections; both are incoherent and rely on nonrobust standards. The first used the two-photon absorption cross-section and fluorescence quantum yield of fluorescein to measure the hyper-Raman cross-section of liquid water.^69^ Despite potential for measuring the hyper-Raman cross-section of simple solvents, this method is not viable for complex systems that may not codissolve with fluorescein, and requires quantitative measurements of the two-photon absorption and fluorescence. The second method used acetonitrile’s hyper-Rayleigh response, which depends upon its static hyperpolarizability, as an external standard.^27^ While this approach is viable for quantifying hyper-Raman response from complex systems, it is not ideal because of difficulties in measuring the static hyperpolarizability.^25^ As a result, quantification of β is often limited to estimates.^25,28^

In a departure from previous approaches, this letter demonstrates how coherent, four wave mixing spectroscopy can extract the hyper-Raman hyperpolarizability. We use the quantitative technique pioneered by Levenson and Bloembergen,^58,59,71^ in which the response of a four-wave mixing process of unknown strength is directly measured against a well-characterized four-wave mixing process present in the same phase-matching geometry, which allows measurement of its nonlinear susceptibility.^59,63,71–73^ In this letter, we extract the hyper-Raman hyperpolarizability of the acetonitrile CN stretch, ν­(CN) (2253 cm^–1^), by measuring its hyper difference frequency generation (HDFG) response against the well-known coherent anti-Stokes Raman spectroscopy (CARS) response from the deuterobenzene ring breathing mode, ν_1_ (944 cm^–1^). The experiment directly extracts the HDFG third-order susceptibility, from which the hyper-Raman hyperpolarizability can then be derived with only knowledge of the ν­(CN) absorptive cross-section.^74,75^ Since all infrared active transitions are hyper-Raman active,^76,77^ this hyper-Raman quantification method is general to all infrared active modes.

To perform four-wave mixing spectroscopy, a Nd:YAG laser (Coherent Infinity 40–100; ∼3.5 ns pulsewidth, 450 mJ at 1064 nm) pumped a dual-line OPO/OPA (LaserVision), generating two tunable and scannable infrared pulses (ω1, ω2) and a second harmonic of the pump (ω_3_ = 532 nm).^78,79^ The ω_1_, ω_2_, and ω_3_ pulse energies were roughly 50 μJ, 500 μJ, and 1.3 mJ, respectively, at a 10 Hz repetition rate. The beams focused at the sample, an acetonitrile/deuterobenzene (91/9 mol %) solution held in a borosilicate rectangular capillary with a path length of 100 μm. The four-wave mixing beam launched in the **k**_4_ = −**k**_1_ + **k**_2_ + **k**_3_ direction was isolated and passed through a double monochromator (SPEX Model 1403; 0.85 m FL) and detected with a photomultiplier tube (EMI 9658R). After preamplification, BOXCAR integration (Stanford Research Systems SR250, 5.5 μs window with 2.3 μs delay), and inversion, the PMT signal was digitized (National Instruments AT-MIO-16E-10). Each data point represents the average of 100 laser shots. The signal was measured as the frequencies of ω_1_ and ω_2_ were scanned across infrared resonances (Figure 2a).^80^ Data workup was conducted using the open-source scientific Python software stack;^81–83^ lineshapes were fit using least-squares optimization. The input and output beams were copolarized so we consider χ_*ZZZZ*_^(3)^, abbreviated henceforth as χ^(3)^ for simplicity.

**Figure 2:**
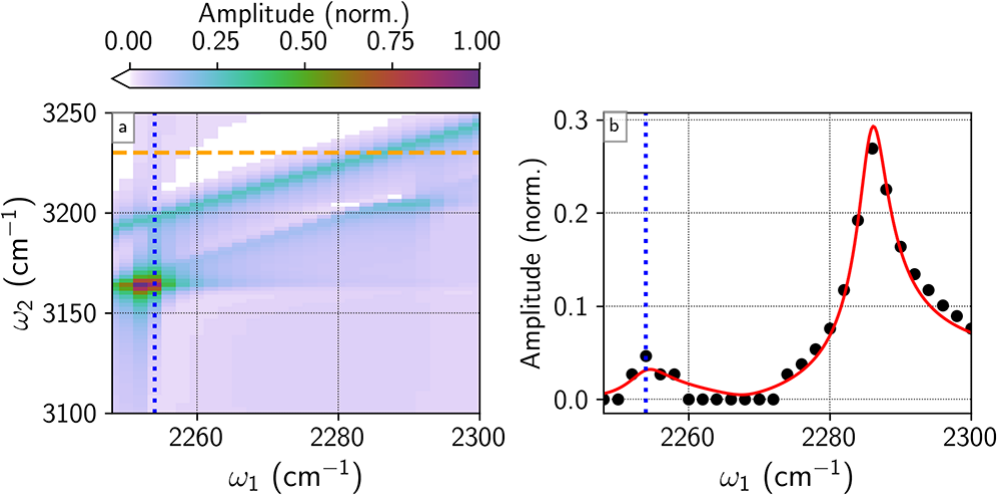
(a) CH_3_CN: 9% C_6_D_6_ four wave mixing response in the **k**_4_ = −**k**_1_ + **k**_2_ + **k**_3_ phase-matching geometry, plotted on the amplitude ( $\sqrt{\mathrm{Intensity}}$ ) scale. For each ω_1_ value, the ω_2_ spectrum is normalized to the C_6_D_6_ CARS response at ω_2_ – ω_1_ = 944 cm^–1^. The dotted blue line indicates the CH_3_CN ν­(CN) frequency. (b) Four wave mixing response (black) and least-squares fit to eq 2 (red) at ω_2_ = 3230 cm^–1^ (dashed orange line in 2D spectrum). The fitting results are found in Table 1. Data points of zero amplitude arise from data below the noise floor of the measurement and do not arise from the data workup scheme used in this work.

**Table 1:** Contributions to the Fit at *ω*_2_ = 3230 cm^–1^ and the Extracted Hyper-Raman Hyperpolarizability, Which Has a ∼25% Error^a^

Process	*A*/Γ	⟨γ⟩ (cm^6^ erg^–1^)	⟨β⟩ (esu)
CH_3_CN HDFG	0.029	2.4 × 10^–38^	3.1 × 10^–32^
C_6_D_6_ CARS	0.244	2.7 × 10^–36^	

a Spectroscopic parameters used to extract ⟨*β*⟩ are the previously reported CH_3_CN *ν*(CN) line width and transition dipole moment,^36^ the C_6_D_6_ *ν*_1_ mode line width,^75^ and CARS hyperpolarizability.^65^

The third order susceptibility of a four wave mixing process is given by

1 $$\chi^{\left(3\right)}=NF\left\langle \gamma \right\rangle$$

where *N* is number density, *F* is the Lorentz local field factor, and γ is the hyperpolarizability; the brackets indicate an orientational average over the spatial degrees of freedom.^84,85^ For a *j* component mixture, the four wave mixing intensity scales with |χ_tot_^(3)^|^2^, where^63^

2 $$\chi_{\mathrm{tot}}^{\left(3\right)}=\chi_{\mathrm{BG}}^{\left(3\right)}+\underset{j}{\sum }\chi_{\mathrm{HDFG},j}^{\left(3\right)}+\chi_{\mathrm{CARS},j}^{\left(3\right)}$$

Response from a doubly vibrationally enhanced (DOVE) process is neglected as its inclusion did not improve fit parameters. For each relevant nonlinear process,^39,75,86^

3a $$\chi_{\mathrm{CARS}}^{\left(3\right)}=\frac{A_{\mathrm{CARS}}}{\omega_{vg}-\left(\omega_{2}-\omega_{1}\right)-i\Gamma_{vg}}$$

3b $$\chi_{\mathrm{HDFG}}^{\left(3\right)}=\frac{A_{\mathrm{HDFG}}}{\omega_{v\prime g}-\omega_{1}+i\Gamma_{v\prime g}}$$

and

3c $$\chi_{\mathrm{BG}}^{\left(3\right)}=A_{\mathrm{BG}}$$

where the prefactors *A*_CARS_ and *A*_HDFG_ depend upon *N*, *F* and ⟨γ⟩ for each species and process.^63^ Like *A*_CARS_ and *A*_HDFG_, the background response (*A*_BG_) is treated as a fitting parameter.^87^ Since the input frequencies are significantly detuned from any electronic resonance in deuterobenzene or acetonitrile, *A*_BG_ is taken to be strictly real.^71^

To accurately extract the hyper-Raman hyperpolarizability, we must address the orientational isotropy of the liquid phase. Under the electric dipole approximation, the HDFG hyperpolarizability of a vibration |*v*′⟩ in a nondegenerate three-beam experiment is^32,34,88^

4 $$\gamma_{ijkl}=-\frac{1}{\varepsilon_{0}}\frac{1}{24\hbar }\frac{1}{\Gamma_{v\prime g}}\beta_{ijk}\mu_{l}$$

where *ε*_0_ is the vacuum permittivity, ℏ is the reduced Planck constant, Γ_*v*′*g*_ is the |*v*′⟩ ⟨*g*| dephasing rate and μ is an infrared transition dipole moment. The lower case letters indicate tensor elements in the molecular frame. Four-wave mixing measurements will isolate the ensemble hyperpolarizability

5 $${\left\langle \gamma \right\rangle }_{IJKL}=-\frac{1}{\varepsilon_{0}}\frac{1}{24\hbar }\frac{1}{\Gamma_{v\prime g}}\langle \beta \mu \rangle_{IJKL}$$

where the uppercase indices denote Cartesian indices in the lab frame. In this work, we focus on the fully copolarized experiment *IJKL* = *ZZZZ*. For an arbitrary IR-active vibration with dipole (μ_*x*_, μ_*y*_, μ_*z*_), the lab frame tensor is given by^85^

6 $${\left\langle \beta \mu \right\rangle }_{ZZZZ}=\underset{i,j\in \left\{x,y,z\right\}}{\sum }\frac{\mu_{j}}{15}\left(\beta_{iij}+\beta_{iji}+\beta_{jii}\right)$$

Depending on the system, the tensor sum in eq 6 may be further simplified by incorporating molecular symmetry or invoking Kleinman symmetry.^89^ Kleinman symmetry is inappropriate for the present work since one of the frequencies used to generate the hyper-Raman hyperpolarizability is near a vibrational resonance.^90,91^ In the gas phase, an isolated CH_3_CN molecule belongs to the *C*_3*v*_ or pseudo *C*_∞*v*_ point group.^92^ However, local structure in liquid phase CH_3_CN complicates its structural description,^93^ rendering the molecular symmetry of *C*_3*v*_ a less adequate representation. Accordingly, we do not apply symmetry relations to simplify the tensor representation.

The HDFG response considered here arises from ν­(CN) of CH_3_CN. By considering the possible symmetries in neat CH_3_CN,^93^ we take the ν­(CN) transition dipole to be aligned along the molecular frame *z* axis,^94^ so that γ_*ijkl*_ will be nonzero only for *l* = *z*. While the presence of C_6_D_6_ may interrupt local structure in liquid CH_3_CN,^95^ it probably does not extensively realign the ν­(CN) transition dipole.^96^ Enforcing this constraint gives^88^

7 $${\left\langle \beta \mu \right\rangle }_{ZZZZ}=\frac{\mu_{z}}{15}\underset{\left\langle \beta \right\rangle }{\underset{︸}{\underset{i\in \left\{x,y,z\right\}}{\sum }\beta_{iiz}+\beta_{izi}+\beta_{zii}}}$$

In eq 7, β terms are factored from the transition dipole μ_*z*_, so we define a new quantity, ⟨β⟩, representing the sum of molecular tensor components the experiment recovers. Finally, by substituting eq 7 into eq 1, we can write the hyper-Raman hyperpolarizability in terms of the susceptibility:

8 $$\left\langle \beta \right\rangle =-\frac{360\hbar \varepsilon_{0}}{NF}\frac{\Gamma_{v\prime g}}{\mu_{v\prime g}}\chi_{\mathrm{HDFG}}^{\left(3\right)}$$

Note that the current polarization scheme, *ZZZZ*, selects specific linear combinations of β_*ijk*_. By considering other viable polarization schemes and taking linear combinations of the resultant outputs,^97,98^ some terms in ⟨β⟩ can be eliminated,^99^ providing stricter bounds when quantifying specific β_*ijk*_ elements. For example, in the case of the ν­(CN) stretch considered here (sum over *i* ∈ {*x*, *y*, *z*} implied), ⟨βμ⟩_*ZZYY*_ - ⟨βμ⟩_*ZZYY*_ = $\frac{\mu_{z}}{6}\left(\beta_{iiz}-\beta_{zii}\right)$ . The difficulty in performing quantitative polarized experiments is choosing a well-described internal standard whose CARS response survives orientational averaging. In the current study, only the *ZZZZ* polarization is considered.

The two-dimensional representation of the four-wave mixing response from the acetonitrile/deuterobenzene mixture (Figure 2a) is consistent with previously reported results. Assignments of the features in the 2D spectrum are reported elsewhere;^79^ of importance to this work: DOVE response from acetonitrile is present at (ω_1_, ω_2_) = (2253, 3164), (2253, 3200) and (2290, 3200) cm^–1^; the diagonal features at ω_2_ – ω_1_ = 944, 918 cm^–1^ are the deuterobenzene ν_1_ mode and acetonitrile ν­(CC) CARS response, and the vertical feature at ω_1_ = 2253 cm^–1^ arises from acetonitrile ν­(CN) HDFG response. While the spectrum could be made quantitative by referencing to the acetonitrile ν­(CC) CARS response, the deuterobenzene ν_1_ CARS response is an ideal standard because there is no reported deuterobenzene DOVE response in the region explored in this study and its CARS susceptibility is well-known.^39,59,63,65,71^ Nevertheless, these features have been well characterized elsewhere,^75^ imploring a quantitative analysis of the two-dimensional spectrum.

To resolve interference between the HDFG and CARS response, a slice of the 2D spectrum at a specific ω_2_ color is taken (black dots, Figure 2b). The line shape of the acetonitrile/deuterobenzene mixture at ω_2_ = 3230 cm^–1^ is fit using |χ^(3)^| and eq 2, with fit parameters found in Table 1. Any ω_2_ frequency could be used for the fitting procedure; 3230 cm^–1^ was chosen to minimize DOVE contributions and maximize the difference between the HDFG and CARS response. In the fit, the vibrational frequencies and dephasing are held as known parameters.^75^ When on resonance, i.e., when ω_1_ = ω_*v*′*g*_ for HDFG and ω_2_ – ω_1_ = ω_*vg*_ for CARS, χ_*k*_^(3)^ reduces to *A*_*k*_/Γ, reported in Table 1 for the *k* ^th^ contribution to eq 2. Taking a ratio of the HDFG and CARS terms that make up eq 1 when on resonance gives

9 $$\frac{\chi_{\mathrm{HDFG}}^{\left(3\right)}}{\chi_{\mathrm{CARS}}^{\left(3\right)}}=\frac{A_{\mathrm{HDFG}}}{\Gamma_{\mathrm{HDFG}}}\frac{\Gamma_{\mathrm{CARS}}}{A_{\mathrm{CARS}}}=\frac{1-x}{x}\frac{N_{{\mathrm{CH}}_{3}\mathrm{CN}}}{N_{C_{6}D_{6}}}\frac{\left\langle \gamma_{\mathrm{HDFG}}\right\rangle }{\left\langle \gamma_{\mathrm{CARS}}\right\rangle }$$

where *x* denotes the C_6_D_6_ mole fraction and *N* is the number density of the pure species at 293 K and standard pressure.^100^ Since C_6_D_6_ and CH_3_CN are in the same solution, their local field corrections roughly cancel. Using the reported deuterobenzene ν_1_ mode CARS hyperpolarizability^39,65^ in eq 9, the number densities of deuterobenzene and acetonitrile at 293 K and 1 atm, and substituting into eq 8 extracts a hyper-Raman hyperpolarizability of ⟨β⟩ = (3.1 ± 0.8) × 10^–32^ esu.

Since the ω_2_ beam that stimulates the hyper-Raman transition was tuned near infrared active modes at ∼3164 and 3200 cm^–1^, the ⟨β⟩ value can be biased by resonant contributions.^101,102^ While these contributions are likely small, they can slightly manipulate the ⟨β⟩ value reported here. DOVE response that involves the transitions at 3164 and 3200 cm^–1^, neglected in the fitting procedure, could also manipulate the extracted value. When DOVE response was included in the fitting procedure used in Figure 2b, it was found that at ω_2_ = 3230 cm^–1^, the DOVE contributions were minimal, consistent with previous simulations on four wave mixing spectroscopy of similar acetonitrile:deuterobenzene mixtures.^75,79^ By performing the extraction procedure for all ω_2_ values in the 3210–3240 cm^–1^ region, the ω_2_ frequencies scanned over in this work where both acetonitrile HDFG and benzene CARS response coexist with good signal-to-noise, the ⟨β⟩ values were found to be consistent within experimental error. As a result, we suspect the extraction is not strongly manipulated by DOVE resonances in the spectrum.

While our extraction of ⟨β⟩ is precise, it is hard to evaluate its accuracy because, to our knowledge, there are no experimental ⟨β⟩ data for the CH_3_CN ν­(CN) vibration. Nonetheless, we can establish some context by comparing this value to the static hyperpolarizability ⟨β_0_⟩ of liquid CH_3_CN. Hyper-Rayleigh scattering measurements extracted a static hyperpolarizability of ⟨β_0_⟩ ∼ 9.8 × 10^–32^ esu at an excitation wavelength of 1064 nm.^27,103^ Theoretical results at the CCSD­(T) level of theory on an isolated, gas phase CH_3_CN molecule found ⟨β_0_ (532 nm)⟩ ≈ 1.36 ⟨β_0_(1064 nm)⟩.^104^ Applying this relation to the liquid phase static hyperpolarizability shows ⟨β_0_(532 nm)⟩ ≈ 1.33 × 10^–31^ esu, roughly a factor of 5 larger than our liquid phase ν­(CN) hyperpolarizability measured at ω_2_ + ω_3_ = 454 nm. A report on hyper-Raman spectra of neat CH_3_CN collected with an excitation wavelength of 1064 nm noted that the hyper-Rayleigh response was two orders of magnitude larger than the reported hyper-Raman response.^12^ Importantly, spontaneous hyper-Raman and hyper-Rayleigh scattering are formally six-wave mixing (χ^(5)^) experiments, and consequently the relationship between β in the lab frame and the molecular frame tensors is different than in HDFG.^105–107^ Particularly, while HDFG response depends upon ⟨β⟩ (eq 6), hyper-Raman and hyper-Rayleigh response depend upon ⟨|β|^2^⟩ and ⟨|β_0_|^2^⟩, respectively.^108^ As a result, the number and weights of various β_*ijk*_ elements in hyper-Raman and hyper-Rayleigh scattering are different than those that contribute to HDFG response.^76,108,109^ A precise comparison will require isolation of specific molecular frame tensor quantities, which is beyond the scope of this work. In any case, these comparisons show that the extracted ν­(CN) hyper-Raman hyperpolarizability value is reasonable.

Similar to the Raman polarizability,^28,46,50^ β has a frequency dependence due to electronic resonances,^27,28,110^ and our extracted ⟨β⟩ value is rigorously correct only for the hyper-Raman excitation ω_2_ + ω_3_ ∼ 22000 cm^–1^. Although this excitation frequency is roughly 55000 cm^–1^ detuned from the lowest lying electronic absorption in acetonitrile (∼77000 cm^–1^),^47,111^ the Raman and hyper-Raman hyperpolarizabilities have a long asymptotic frequency dependence from these electronic states.^50^ Understanding these dispersions are relevant for frequency-dependent resonance hyper-Raman studies that investigate excited state structure.^28^ To quantify resonance hyper-Raman response of a molecule with an unknown hyper-Raman response at different excitation frequencies, the frequency dependence of the solvent hyper-Raman response, the internal standard, must also be quantified. Performing quantitative four wave mixing with a tunable visible source and using an internal standard with an established Raman cross-section dependence can map out the frequency dependence of ⟨β⟩, and rigorously enable quantitative, frequency-dependent resonance hyper-Raman spectroscopy. While the measurements herein were performed with a nanosecond laser system that does not have a tunable visible source, quantitative four-wave mixing spectroscopy has been extended to ultrafast spectrometers with tunable and scanable infrared and visible sources,^66,112,113^ which will be useful for extending the measurements conducted here.

Beyond enabling quantitative hyper-Raman spectroscopy, which could become a valuable probe of excited state structure,^25,28,30^ the ⟨β⟩ measurement provides other useful constraints in both the theoretical and experimental realms.^25,32^ Calculating hyper-Raman cross-sections from first-principles is notoriously difficult.^25,92,108,109,114–116^ When calculating the hyper-Raman cross-section, a static hyperpolarizability and its geometric derivatives must also be calculated, both of which are highly sensitive to the level of theory used in calculations.^109,114,117^ Measuring the frequency dependence of ⟨β⟩ for key vibrational modes can provide strict experimental constraints for improving first-principles β_*ijk*_ and hyper-Raman cross-section calculations. Quantitative ⟨β⟩ values can also be used as internal standards in spontaneous and coherent hyper-Raman processes and to predict nonlinear processes involving hyper-Raman transitions. These values are essential for performing preresonant, low-concentration hyper-Raman spectroscopy and promoting further developments in nonlinear and also entangled-photon spectroscopy.^32,118^

In summary, quantitative four-wave mixing spectroscopy was used to measure the preresonant hyper-Raman hyperpolarizability of the acetonitrile CN stretch, ν­(CN). Applying this approach to other systems should provide a general way to quantify hyper-Raman hyperpolarizabilities. Measuring hyper-Raman hyperpolarizabilities of multiple vibrations will firmly cement hyper-Raman based spectroscopies as quantitative techniques, which could become useful tools for investigating excited state surfaces and vibronic coupling schemes inaccessible with Raman transitions. By having a method to routinely measure hyper-Raman hyperpolarizabilities, the feasibility of nonlinear spectroscopies involving hyper-Raman transitions can be further explored and spontaneous hyper-Raman experiments, whether in a resonance or preresonance regime, can become firmly quantitative.

## Data Availability

The data and workup scripts that support this work are found on the Open Science Framework (OSF) at 10.17605/OSF.IO/WS2YU.
